# Drug Target Commons 2.0: a community platform for systematic analysis of drug–target interaction profiles

**DOI:** 10.1093/database/bay083

**Published:** 2018-09-13

**Authors:** ZiaurRehman Tanoli, Zaid Alam, Markus Vähä-Koskela, Balaguru Ravikumar, Alina Malyutina, Alok Jaiswal, Jing Tang, Krister Wennerberg, Tero Aittokallio

**Affiliations:** 1 Institute for Molecular Medicine Finland (FIMM), University of Helsinki, Helsinki, Finland; 2 Department of Mathematics and Statistics, University of Turku, Turku, Finland; 3 Biotech Research & Innovation Centre (BRIC), University of Copenhagen, Copenhagen, Denmark

## Abstract

Drug Target Commons (DTC) is a web platform (database with user interface) for community-driven bioactivity data integration and standardization for comprehensive mapping, reuse and analysis of compound–target interaction profiles. End users can search, upload, edit, annotate and export expert-curated bioactivity data for further analysis, using an application programmable interface, database dump or tab-delimited text download options. To guide chemical biology and drug-repurposing applications, DTC version 2.0 includes updated clinical development information for the compounds and target gene–disease associations, as well as cancer-type indications for mutant protein targets, which are critical for precision oncology developments.

## Introduction

Accurate identification of interactions between ligands and target proteins is a key prerequisite for understanding the biological action of chemical tool compounds and drugs. With the constant accumulation in the number and diversity of biological and chemical assays, an ever-increasing amount of quantitative data on compound–target interactions is available in the primary literature and public databases. This data can be used for discovery of new indications for drugs, i.e. drug-repurposing (1) or for selection of compounds targeting specific proteins or pathways of interest (2). The drug–target interaction data are typically in the form of biochemical affinity measurements but may also include quantitative structure–activity relationships, which can be used for computational models predicting compound–target interactions and extended target spaces for drugs for which no target interaction data are currently available, i.e. predictive drug positioning (3, 4, 5). Integration with chemical proteomic data can refine drug-affected pathways, identify response markers and suggest novel combination treatments (6). These data resources and models may also be useful for predicting drug side-effects, *in vivo* absorption, distribution, metabolism, excretion and toxicity properties (7). After validation steps, the observed drug phenotypic effects can also be used to improve the accuracy of computational models (8).

Several databases have been implemented for providing open access to compound/target information. These can roughly be categorized based on the type of molecules or assays covered and their end purpose. Below, we will provide short overview of the related key databases. Examples of databases providing broad target and drug information include ChEMBL (9) and PubChem (10), which list bioactivities of drugs and drug-like small molecules extracted either from the scientific literature or generated through high-throughput screening experiments. DrugBank (11) combines detailed drug information (i.e. chemical, pharmacological and pharmaceutical) with comprehensive target information (i.e. sequence, structure and pathway). BindingDB (12) contains binding affinities for small drug-like molecules, and GtopDB provides information about structures for small molecules, peptides and antibodies with their affinities for protein targets (13). Yet other databases have been geared toward linking drug–target data to genetic information, in one aspect for predicting phenotype based on genotype. As an example, DGidb (14) uses a combination of expert curation and text mining integrated from DrugBank, Therapeutic Target Database (15) and PharmGKB (16) to document putative drug–gene interactions.

On the other hand, some databases function not only as information repositories but also serve to facilitate research by functioning as query portals or visualization tools for biological questions. For example, Chemical Probes (17) is a recent community-driven web application that recommends appropriate chemical probes for biological targets, provides guidance on their use and documents their limitations. Probe Miner (18) implements Chemical Probes Objective Assessment resource, capitalizing on the plethora of public medicinal chemistry data to empower quantitative, objective and data-driven evaluation of chemical probes. STITCH (19) is a comprehensive resource to explore and visualize experimentally tested and computationally predicted interactions among chemicals and proteins, which helps researchers identify and position their favorite molecules in complex biological systems. LINCS (20) aims to create network-based understanding by cataloguing changes in gene expression and other cellular processes in response to a variety of perturbing agents. DrumPID (21) provides researchers with tailored information on drugs and protein interactions and enables one to screen related compounds for their effects on protein interaction networks considering data also from other organisms. The iHOP (22) web server provides up-to-date summary information on biological molecules by automatically extracting key sentences from millions of PubMed documents. Finally, Open PHACTS integrates data from multiple publicly available databases, such as ChEMBL, DrugBank, ChEBI, UniProt and WikiPathways, to enable researchers to build pipelines based on integrated pharmacological data resources (23).

While the aforementioned resources have been useful for phenotypic profiling and drug development efforts, they provide only a limited assay annotation for the end users to understand and sort out the variability in the bioactivity data that are generated using various assays, resulting in significant heterogeneity and potential discrepancy between the databases (24). ChEMBL is currently the most comprehensive, manually curated database, consisting of compound–target bioactivity values for over 1.8 million compounds. However, comprehensive extraction and annotation of compound–target bioactivities is a tedious process, beyond the capability of a single team or institution. Toward this end, we recently introduced a community-driven bioactivity collection and standardization platform, named Drug Target Commons (DTC), which includes a user-friendly web interface and a simplified bioassay ontology (μBAO) (25). In the present report, we describe the technical implementation and recent updates of the DTC database and its web interface. Foremost, we have vastly increased the number of annotated data points (~16 000 bioactivities) and integrated ~0.5 million additional published bioactivities from the BindingDB (12). The extended DTC version also includes clinical development information for the compounds as well as target gene–disease associations and cancer-type indications for mutant targets, which should be highly useful for translational research and basic molecular understanding alike. Lastly, we have made several updates to the web interface, which should further lower the user threshold and improve the attractiveness of DTC.

## User interface

Key features for end users include options to search, filter, sort, import, edit, export and manage bulk bioactivity data and associated information for compounds and targets in a user-friendly manner. Drug lists are easy to filter and users always have the option to download only parts of the database or all of it. On the other hand, any new annotations or modifications on existing data are first subjected to review by the group of administrators before depositing as entries in DTC. Log-in is easy via Google account and mandatory for data curation and uploading of new data. Since the review of annotations as a quality control is critical for maintaining the accuracy and reliability of the database, researchers may request to become administrators to facilitate/accelerate data uptake into DTC. Approved administrators are notified by email as soon as sufficient amount of newly deposited data enters into the DTC system.

### Data download options

The full bioactivity data in DTC are available for downloading in tab-delimited text format on the download tab (http://drugtargetcommons.fimm.fi/static/Excell_files/DTC_data.csv). There are currently ~6.5 million bioactivity data points (for protein targets), which are available for research purposes under the Creative Commons license (CC BY-NC-SA 3.0). Complete database dump as well as application programming interface (API) is provided to facilitate an easy access and integration of data with scripting tools. Detailed description for API is available on the download tab at DTC website. Entity relationship diagram (see online supplementary material for Supplementary Figure S1) provides users with a detailed understanding of the database schema. Several search options are available in the graphical user interface (GUI) to enable downloading only selected sets of bioactivity data (see below).

### Compound and target search options

**Figure 1:**
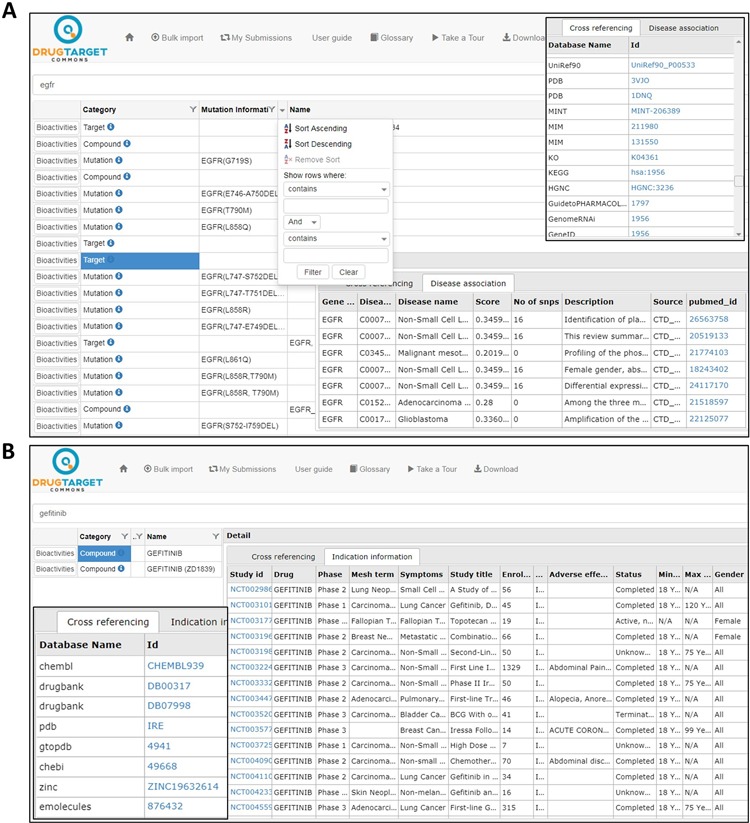
Search results for compounds and targets. (**A**) Gene–disease associations and cross-referencing information for target EGFR. (**B**) Cross-referencing and clinical trial information for compound gefitinib.

Bioactivity data in DTC can be searched using a variety of compound and target identifiers (see online supplementary material for Supplementary file S1), PubMed ID (for the publications) and somatic mutation information (e.g. D835Y mutation in the *FLT3* gene). DTC compounds are cross-linked with 25 different databases (e.g. DrugBank, PubChem, ChEMBL and PharmGKB) using compound ID-mapping data and 94 different protein target databases (e.g. UniProt, Ensembl, EMBL, PDBe, HGNC and Uniref) using target ID-mapping data (see online supplementary material for Supplementary file S1). Compound ID-mappings were obtained from UniChem (https://www.ebi.ac.uk/unichem/info/downloads), and target ID-mappings from Uniprot (ftp://ftp.uniprot.org/pub/databases/uniprot/current_release/knowledgebase/idmapping/). An autosuggest feature was implemented to facilitate end users in selecting the appropriate search item. Help options for search identifiers can be seen by clicking at ‘?’ next to the ‘search textbox’ (Figure 1A).

Search results may include any of the following categories: target, compound, publication or mutation (see Figure 1A). Cross-references to other databases are shown by clicking at the ‘info icon’ in front of respective search category; these include, for instance, gene–disease associations from DisGeNet for target genes and clinical phase development information for compounds (Figure 1B).

### Editing and filtering options

**Figure 2:**
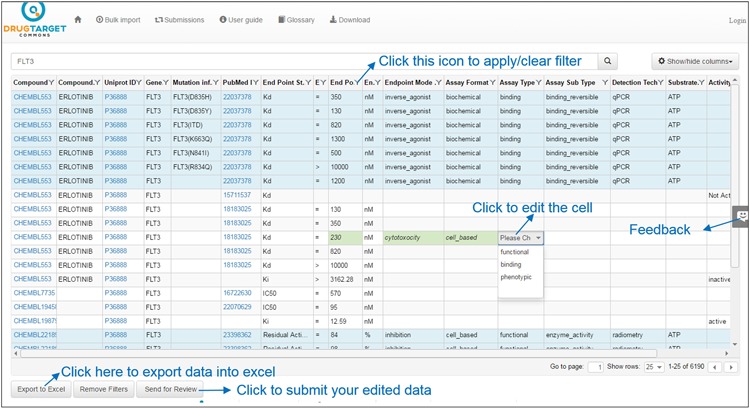
Bioactivity data values for target FLT3. Light blue background shows the annotated bioactivity values, whereas white background shows unannotated bioactivity data.

Users may view or suggest edits on bioactivity data by clicking on the ‘Bioactivities’ button. Sorting is done by clicking at the column header and multiple columns can be sorted (similar to Excel) by clicking the headers. Users may filter each column of the table by clicking the filter icon next to column header and then applying filter conditions on column from the variety of available filter types. Filter types for string data are Contains, Does not contain, Starts with, Ends with, Empty and Not empty and for numeric data Equal, Not equal, Less than, Greater than, Null and Not null. Filtering conditions can be merged using ‘OR’ and ‘AND’ operators. Filtering options are case-insensitive (i.e. GEFITINIB and gefitinib are the same). Users may also remove filter condition by clicking on the ‘Clear’ button at the bottom of filter options as shown in Figure 2.

### Bioassay annotations and cross-linking

DTC is linked to over 25 other databases from which the affinity, IC_50_ and other bioactivity values are being obtained. For the annotation part, we adapted a so-called μBAO protocol, which is a simplified version of the original BAO (26) that standardizes the description of target-profiling experiments in terms of the assay type, assay format, endpoint type, detection technology and inhibitor types. In μBAO, we included only those assay annotation fields that we consider as minimum set of required information to describe a bioactivity experiment and are likely to be extractable from research publications, as explained in our recent publication (25). Such a standardized bioassay annotation should improve the understanding and consistency of the bioactivity data in DTC, and therefore be critical for data interpretation. Equally important for the community-based aspect of DTC is that data annotation should be as smooth and streamlined as possible for annotators, where μBAO is a step toward simplifying the process. A user feedback form available on the website and emailed questionnaires help to shape DTC into a user-friendly experience as possible.

The DTC GUI provides hyperlinks to the reference publications in order to cross-check bioactivity data and to annotate assay information. The μBAO annotations can be selected from the drop-down options in the user interface. Explanation of each μBAO term is provided in the ‘Glossary’ under the ‘Help’ tab. To enable multi-record editing, ‘copy/paste’ in data tables is permitted to speed-up editing. After making the relevant modifications, users may click at ‘Send for review’ to submit suggestion for review. Users can see (as well as modify) their submissions at ‘My Submissions’ tab (https://drugtargetcommons.fimm.fi/submissions/), which holds a temporary copy of the submissions until ‘Approve or decline’ decisions are made by administrators of DTC. Administrators may further modify the submissions prior to approval. Upon approval by the administrators, the relevant submission is integrated into the DTC databases and can be viewed in the next search queries. To avoid the problem faced if administrators were approving duplicate submissions, we wrote Cron-scripts to process duplicated or unwanted data prior to administrator’s view. Cron-scripts are automatic scripts scheduled to repeatedly execute after a fixed period to assess the quality (pre-processing) and remove redundancy from the newly submitted data.

### Export and import in Excel

After sorting or filtering, bioactivity data can be exported to Excel (by clicking on ‘Export to Excel’ button), as shown in Figure 2. There may be missing information for some of the columns, depending on the annotation status at the time of exporting. On the other hand, bulk data can also be curated in an offline mode in Excel and later uploaded back to DTC (a template file is provided at ‘Bulk Import’ page). Users may also view, modify, filter and sort newly uploaded file through DTC interface, and once satisfied, submit their data for review. DTC administrators will be notified via automatic emails to process the newly uploaded data (after the quality control by Cron-scripts).

### Disease–target associations

**Figure 3:**
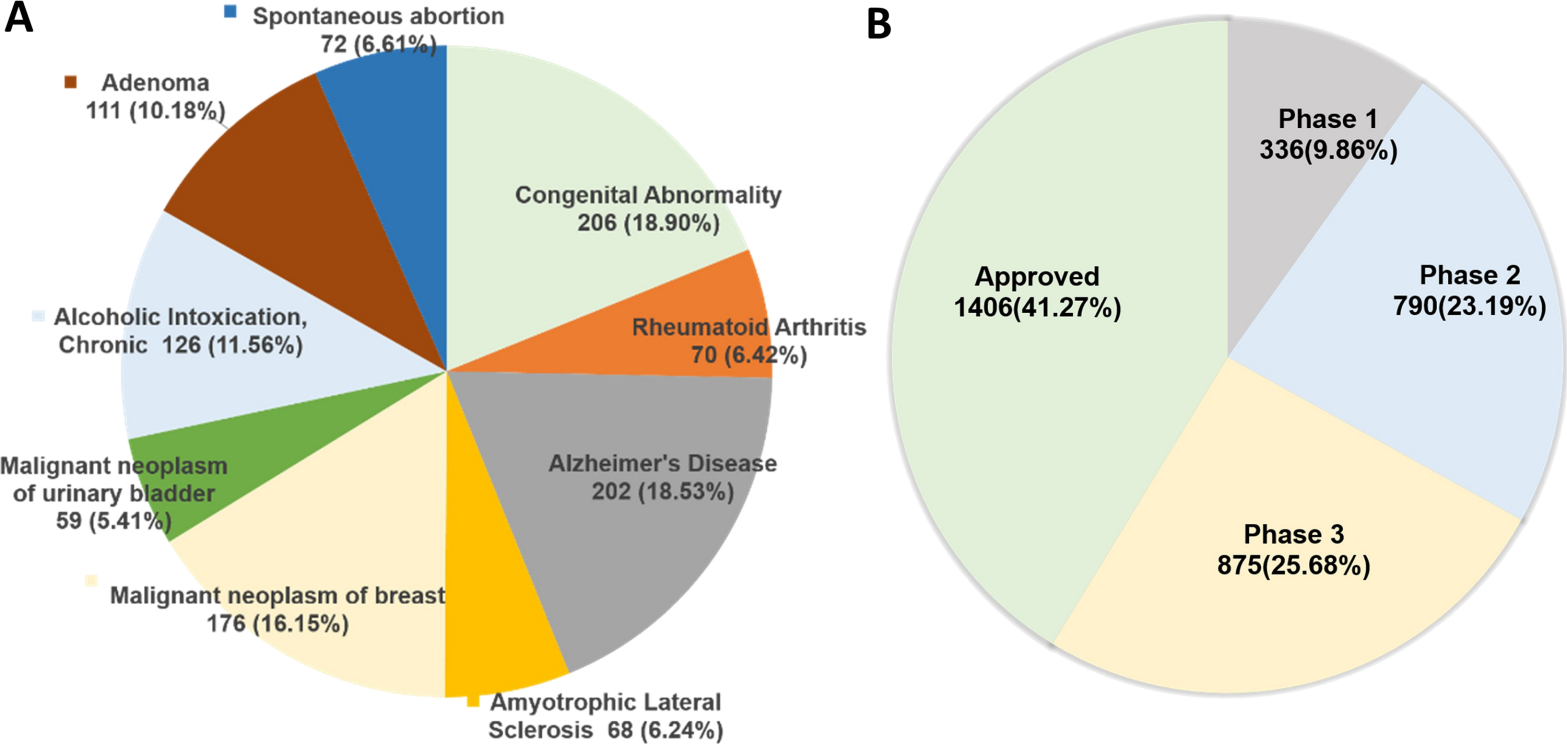
(**A**) Protein targets associated with diseases extracted from DisGeNET (only top 10 diseases for the current DTC targets are shown here; see online supplementary material for Supplementary File 3 for the full list). (**B**) Highest clinical phase for 3407 DTC small molecules that have information in clinical trials database (http://clinicaltrials.gov/). The approved category includes 1406 compounds, which are also overlapping with the Santos *et al.* drug list.

For the protein targets in DTC, curated gene–disease associations are extracted from DisGeNET (27). There are currently 1573 genes associated with 4123 diseases having 331 514 associations supported by references (top 10 diseases are shown in Figure 3A). Cancer-type indications for 185 mutant protein targets are extracted from Cancer Genome Interpreter (CGI) (28), supported by clinical evidence. This information can be accessed through DTC search page (by clicking ‘info’ icon).

### Clinical development information

As a recent addition to the DTC system, we extracted up-to-date clinical development information for 3532 compounds (292 218 indications), including both approved drugs and investigational compounds currently undergoing clinical trials from https://clinicaltrials.gov/. Figure 3B shows the distribution of DTC compounds across different clinical phases. The clinical information in DTC includes the following: study details, compound name and development phases, symptoms, mesh terms, adverse effects, participants’ information, eligibility criteria, reference publications, as well as references for the clinical study. This information can be accessed by clicking ‘info icon’ in front of a searched compound (Figure 1B). We believe this information will become highly useful for drug-repurposing applications, where the aim is to find novel uses of already-approved drugs or those in the later stages of clinical development.

### Crowd-sourced curation

As DTC is expected to attract a large amount of contributions submitted by variety of users, and later subjected to the processing by an expert panel, there is a need to systematically deal with different categories of users. Crowdsourced curation in DTC is systematized by defining four user groups: super administrators, administrators, trusted curators and other users. Super administrators (currently the developers of DTC) can approve the status of administrator for any user (applied through https://drugtargetcommons.fimm.fi/admin/ or by email). The administrators and super administrators act like reviewers and process the submitted bioactivity data as well as to approve a user for ‘trusted curators’ group. Each user group has certain permissions, which can be altered by the super administrators. The rationale for distinguishing ‘trusted curators’ from ‘other users’ is to provide a flag for the administrators to pay particular attention to the submissions by the new and potentially unexperienced ‘other users’.

Curators can upload newly curated and annotated data through bulk upload feature as explained in Section 2.5. See online supplementary material for annotation and curation guidelines that are available in Supplementary File 4 and at the ‘Help’ tab (http://drugtargetcommons.fimm.fi/annotation_guidlines/). These guidelines follow a curation standard developed in-house, based on experience from similar curation tasks (26, 29). Previous user feedbacks are publicly available for the new users who can see the comments by the previous users and our responses to those comments. A curator can annotate data points for compound(s), target(s) or publication(s), but we recommend performing annotations publication-wise as this reduces workload for the annotators (often the same assay type is used for all data in a single publication). In addition, we advise new curators to look at the ‘Take a Tour’ tab on the DTC website, in order to quickly familiarize with the overall DTC workflow.

Currently, DTC curation team is comprised of around 15 in-house researchers, including cell biologists and data scientists, who are working as a core data curation and annotation team, and are assigned to non-overlapping compound to perform μBAO annotations in addition to the curation of new compounds/targets. However, DTC effort is open to anybody, who wants to be part of the DTC annotation team. We are storing the annotator’s identity that can also be publicly shown to others (upon annotator’s request), along with the deposited bioactivity data points. In addition, we give authorship in the new releases of DTC to all the significant contributors (data curators and annotators). Inconsistencies between curators are sorted out by the administrators, and only after the administrator’s approval, the curated and annotated data are systematically integrated back into DTC. The resulting resource of annotated and curated data is freely available to the DTC crowdsourcing team, as well as to the whole chemical biology community.

## Data sources

ChEMBL is currently the main source of bioactivity data in DTC, which are further validated by DTC curation team and annotated using the μBAO annotations. Additionally, we have ~60 000 fully annotated bioactivity values, which are not included in the current releases of ChEMBL or BindingDB but were directly extracted from scientific publications. We have so far completed the annotation of 204 901 bioactivity data points among 4276 chemical compounds and 1007 distinct protein targets. The current annotation process has mainly focused on kinase inhibitors, due to their importance in anticancer drug development; however, the unannotated bioactivity data already stored and searchable in the DTC database span a wide spectrum of compound and target classes. In addition to several in-house annotation and test rounds, we have carried out two user studies, one national (30) and another in European-wide MedBioinformatics Horizon 2020 project (http://www.medbioinformatics.eu), and have improved the DTC platform based on the user feedback.

## Data coverage

**Figure 4:**
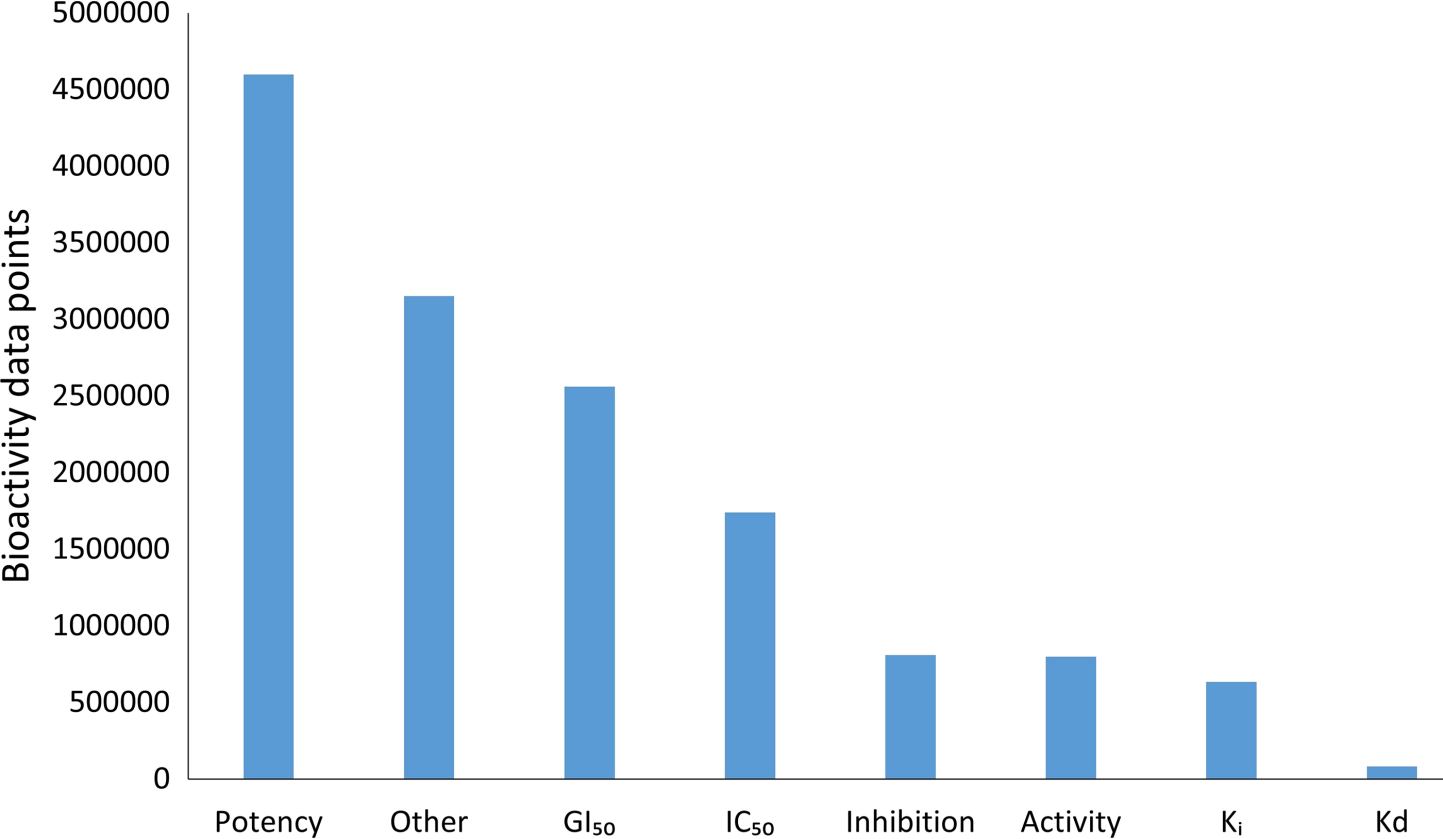
Bioactivity endpoints for the compound–target pairs present in the current DTC version. Bioactivity types (e.g. EC50, XC50, AC50, etc.) with relatively small proportions are grouped under ‘Other’ category.

**Figure 5:**
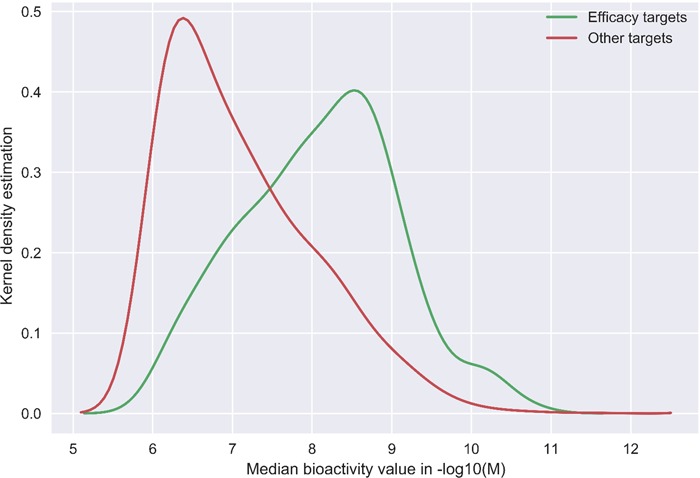
Kernel density plots comparing the DTC bioactivity levels of so-called efficacy target with other targets of 1406 approved drugs from Santos *et al.* drug list (31). In case of multiple bioactivities measurements, the median was taken for a drug–target pair. Potency threshold of 1000 nM was applied to the median bioactivity value and negative log was taken for bioactivity values in molar concentrations.

Although the community-based crowdsourcing and annotation work have just initiated, there exist extensive bioactivity data, across multiple bioactivity endpoints (Figure 4), waiting to be annotated (1 746 997 million compounds, 13 023 targets and 14 820 874 million bioactivities). To evaluate the DTC bioactivity data relevance for drug discovery, we compared density plots for approved drugs in terms of their efficacy targets (31) and other potent targets as shown in Figure 5. For this analysis, we chose a cutoff of 1000 nM for the median bioactivity value, but similar results are obtained also with other potency cutoffs, suggesting that there are also many off-target potencies among the other targets beyond the known efficacy targets of the 1406 approved drugs present in DTC. These could be potential leads to novel drug-repurposing applications. Similar analysis was performed for BindingDB and GtopDB, as shown in Figure S2 and S3, respectively (see online supplementary material for both figures). For that purpose, we downloaded data from BindingDB and GtopDB and matched their compounds with the approved drugs using standard InChiKeys. For the target comparisons, we used UniProt ID as an identifier. There are 917 and 641 approved drugs from Santos *et al.* (27) present in BindingDB and GtopDB, respectively. For these drugs, at least 7% of the off targets in BindingDB have concentration <1 nM, which could provide possible candidates for drug-repurposing (see online supplementary material for Figure S2). Similarly, a fraction of off-target potencies present in GtopDB could provide starting point for drug-repurposing applications (see online supplementary material for Figure S3). Analysis performed in Figures 5, S2 and S3 shows the significance of off-target bioactivities included in these databases that store quantitative bioactivity data. However, DTC has numerical superiority both over BindingDB and GtopDB not only in terms of relatively larger collection of approved drugs but also in terms of their associated off-targets.

**Figure 6:**
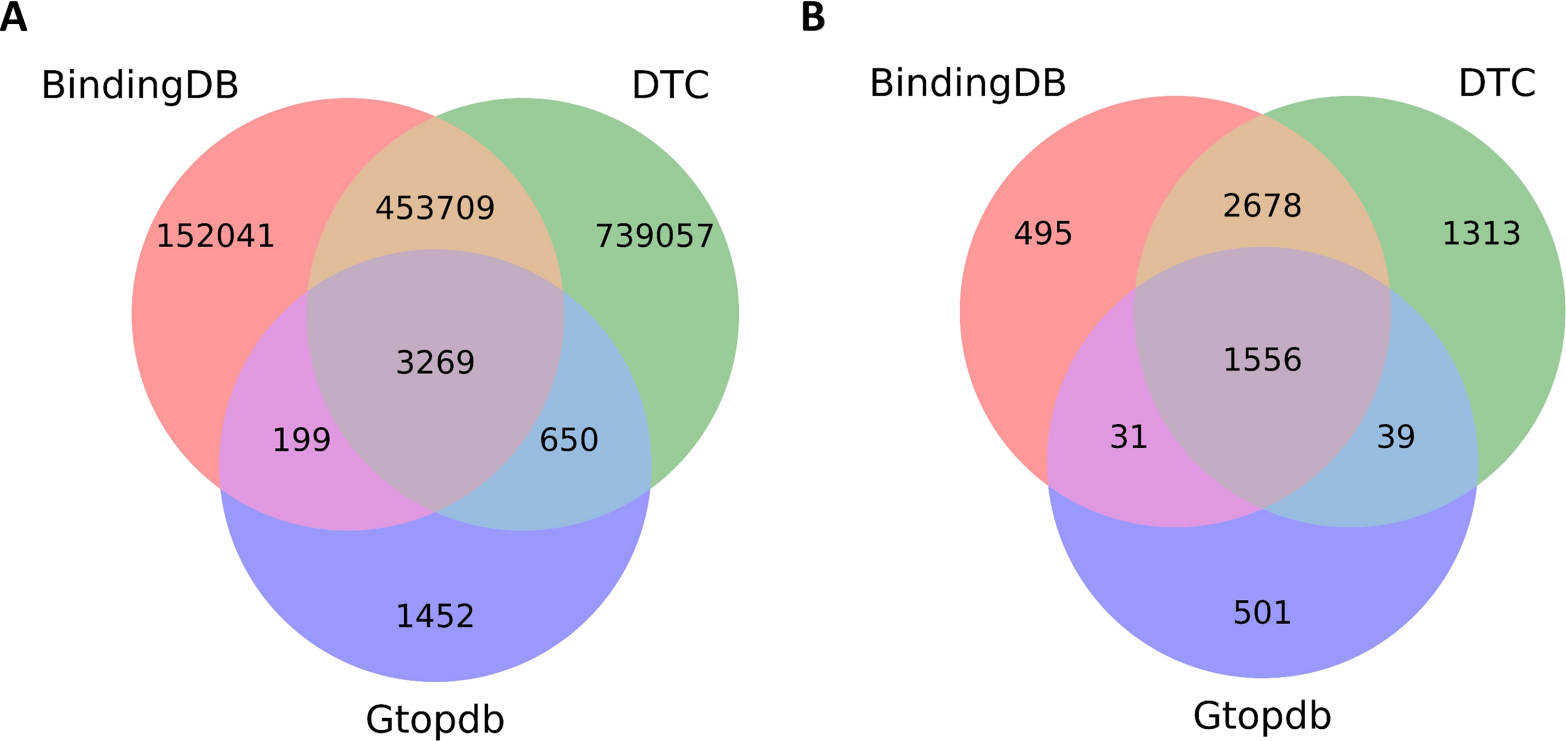
Overlapping compounds and targets between DTC, BindingDB and GtopDB among compound–target pairs for which dose–response measurement (e.g. Kd, Ki and IC50) bioactivity data are present in the databases. (**A**) Overlapping compounds by comparing Standard InChiKeys. (**B**) Overlapping targets by matching UniProt IDs.

To give further insights into compounds and target coverage and overlap, we compared DTC with other bioactivity databases, such as BindingDB and GtopDB. BindingDB and GtopDB contain only dose–response endpoints (K_d_, K_i_, IC_50_ and EC_50_), and likewise in DTC we have considered this data more relevant for biological activity and have therefore excluded from this comparison any single-concentration measurements (activity %, inhibition % and others), which are more prone to technical variation. Furthermore, only the molecularly targeted agents were used in these analyses. The comprehensiveness of the data present in DTC can be seen in Figure 6, which shows significant fraction of non-overlapping compounds and targets in comparison with BindingDB or GtopDB.

## API to access to bioactivity data

API is a specific sub routine to provide programmatic data access to the developers for building their own applications. We implemented API for DTC users to access bioactivity data queried using compound, target or publication information. Output data are returned in XML/Json format and users may apply certain filters to extract subsets of data. The default limit for the output bioactivities is 20, but this can be modified by the user. User can access maximum of 1000 bioactivities at a time, but it is also possible to extract all the bioactivities in DTC by changing the ‘Offset’ parameter. Table 1 lists some examples of the commands that can be used to programmatically access DTC in Python (or any other scripting language), using ‘Curl’ command (note: there should not be space anywhere in URL). A detailed documentation for the API is provided in Supplementary File 2 (see online supplementary material for this file).

**Table 1:** Bioactivity data extraction through API

**Web link**	**Description**
https://drugtargetcommons.fimm.fi/api/data/bioactivity/?filter_field1=FILTER_VALUE1&filter_field2=FILTER_VALUE2	Field name can be compound ID, target ID, mutation information, Pubmed ID, assay format, assay type, etc. Field value is case-sensitive.
https://drugtargetcommons.fimm.fi/api/data/bioactivity/?mutation_info=FLT3(D835Y)	Outputs bioactivity data associated with **D835Y** mutation in **FLT3** gene.
https://drugtargetcommons.fimm.fi/api/data/bioactivity/?detection_technology=qPCR&molecule_chembl_id=CHEMBL939	Outputs bioactivity data associated with detection technology **qPCR** and compound ID **CHEMBL939.**
https://drugtargetcommons.fimm.fi/api/data/bioactivity/?assay_sub_type=enzyme_activity	Outputs bioactivity data associated with assay sub type **Enzyme activity.**
https://drugtargetcommons.fimm.fi/api/data/bioactivity/?molecule_chembl_id=CHEMBL939&limit=100	Outputs maximum of **100** bioactivity data points associated with compound ID **CHEMBL939.**

## Technical implementation

The front end of DTC user interface is implemented in JavaScript, JQuery-1.11 and Bootstrap3.0, whereas the back end is implemented in Python3.5 using Django1.9 framework, which is an open-source framework for Python that supports rapid web development and pragmatic design. For data visualization, we have used JavaScript libraries, such as Amcharts (https://www.amcharts.com/) and D3 (https://d3js.org/), whereas tabular data representation is performed using jQWidgets (http://www.jqwidgets.com/) and JQuery data tables (https://datatables.net/). The first release of DTC was developed in C#.net, which was later replaced with Python, as it is a more popular scripting language for academic researchers.

The DTC database is developed in PostgreSQL 9.0. DTC database is divided into five main categories: compounds, proteins, diseases, assays and activities and others. A detailed entity relationship diagram for the DTC schema is shown in Supplementary Figure S1 (see online supplementary material for the figure). Entity relationship diagram and DTC schema dump are available at the download tab on the DTC website. Indexing is introduced in database tables to reduce search time for Structured Query Language (SQL) queries, and the underlying load on database was further reduced by improved performance using custom caching-based solution on the top of the standard Django cache.

## Application use cases

### Anticancer drug repositioning

DTC contains potent bioactivity data for many protein mutations, which have been implicated in different tumor types. Literature evidence for such protein–disease associations for the mutant targets was extracted from CGI (28). CGI identifies somatic mutations that are known to affect the response of anticancer therapies according to several levels of clinical or preclinical evidence. Here, we present a selected set of DTC-based findings for mutant targets having strong affinities with compounds overlapping with CGI in various tumor types. The syntax for mutant protein targets in DTC is ‘Gene-name(mutation)’, whereas CGI accepts mutations in the following format: ‘Gene-name:mutation’; for instance, *FLT3*(D835Y) and *FLT3*:D835Y, respectively. Table 2 lists representative examples of DTC-based potencies for mutant targets that have strong (median) binding affinities with the listed drugs as supported by clinical evidence. Especially interesting are those cases where the bioactivity for the mutant target is much lower (stronger) than for the wild-type target, as these might provide targeted treatment options for cancers driven by the specific mutation and not severely toxic in the wild-type tissues.

**Table 2:** Examples of DTC-based potencies for mutant targets

***Somatic*** ***mutation***	***Drug name***	Tumour type^*^	***Median bioactivity for mutant target (nM)***	***Median bioactivity for wild type target (nM)***	***Min bioactivity (nM)***	***Max tested bioactivity (nM)***	***Evidence level from CGI***	***Reference***
FLT3 (D835Y)	Midostaurin	AML	15	12	2	10 000	Phase II	(32)
	Sorafenib	AML	82	30	0.021	50 000	Early trials	(33), (34)
ABL1 (T315I)	Axitinib	CML	2.55	60	0.1	10 000	Early trials	(35)
	Crizotinib	ALL	11	103.5	0.55	22 840	Preclinical	(36)
KIT (L576P)	Dasatinib	AML	0.57	3.85	0.016	715 000	Case report	(37)
	Imatinib	GIST	14	219	0.7	15 × 10^9^	FDA guidelines	(38), (39), (40), (41)
	Nilotinib	GIST	22	37.5	1.1	50 × 10^5^	Early trials	(42)

^*^AML: Acute myeloid leukemia, GIST: Gastrointestinal stromal tumors, CML: Chronic myelogenous leukemia and ALL: Acute lymphoblastic leukemia

^*^Min and Max indicate the minimum and maximum bioactivity for a compound across all the targets (wild type or mutant proteins) in DTC

### Web tools that are built on DTC database

#### MediSyn

**Figure 7:**
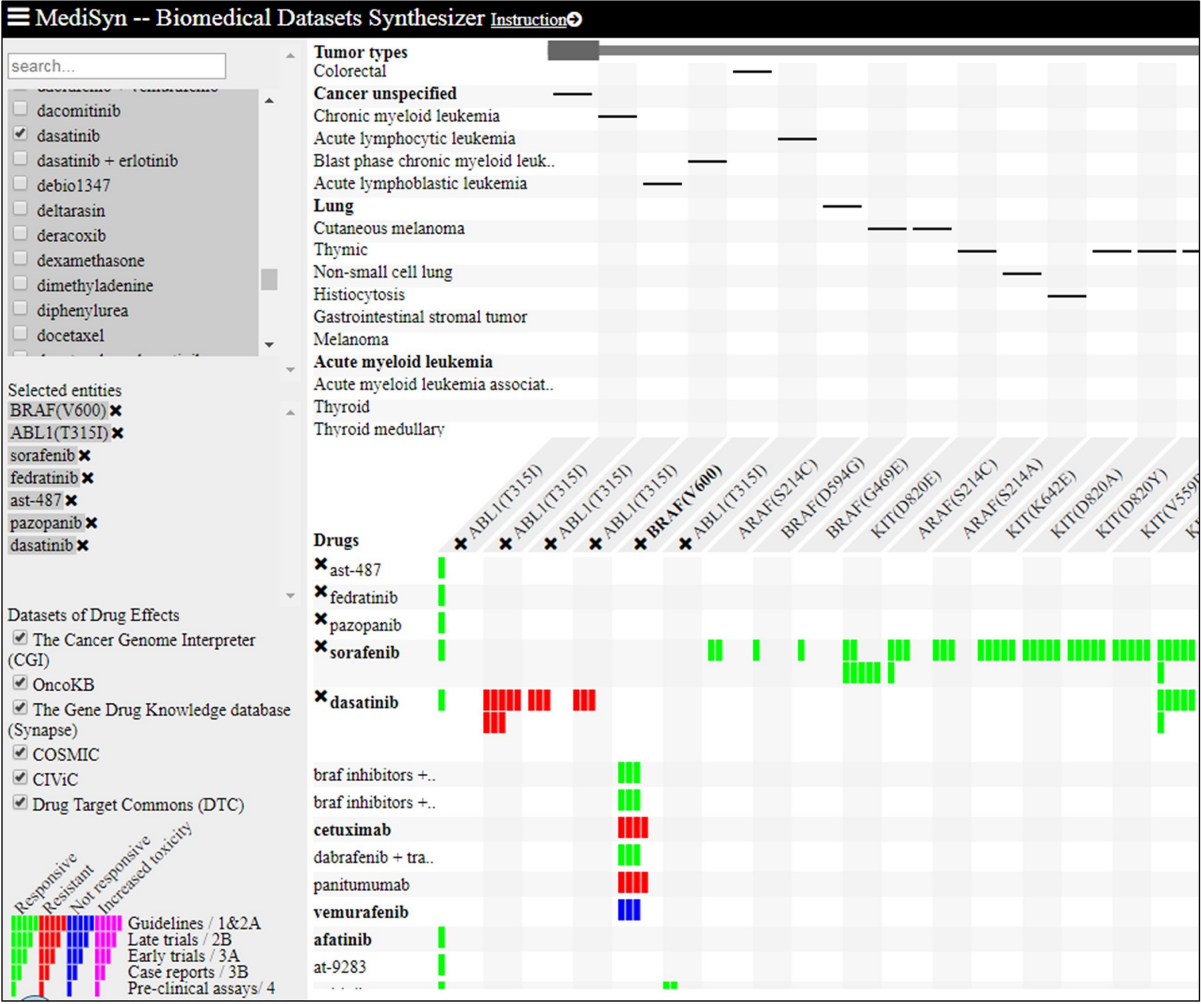
Matrix-based visualization generated by MediSyn for mutants ABL1(T315I) and BRAF(C600E) and compounds AST-487, fedratinib, pazopanib, sorafenib and dasatinib. Compounds are placed at rows, whereas columns contain the associated mutant targets. Green bars represent the responsive compounds, whereas the red bars represent resistant compounds. The number of bars represents different categories of evidences as shown in the legend (bottom left corner). Singleton bars show the preclinical data integrated from DTC.

 MediSyn (https://d4health.hiit.fi/) is a recently introduced web tool that synthesizes multiple medical datasets, including DTC, with the aim to support drug-treatment selection (30). MediSyn uses a matrix-based layout to visually link drugs, targets (including somatic mutations) and tumor types across different datasets using five levels of evidences as shown in Figure 7. Data uncertainties are salient in MediSyn; for example, (i) missing data are exposed in the matrix view of drug–target relations and (ii) data credibility is conveyed through links to data provenance. In the current version of MediSyn, bioactivity data for ~200 unique mutant proteins are extracted from DTC using API in order to extend options for drug-treatment selection. To the best of our knowledge, DTC is the only data source in MediSyn that is providing preclinical evidences (represented by single bars in Figure 7), combined with μBAO and compound clinical phase information. Moreover, based on DTC bioactivity data, MediSyn also gives extra hits, especially for the compounds that are not yet in clinical use. For instance, AST-487 and fedratinib both target ABL1(T315I) mutant and are currently undergoing clinical trials. Similarly, pazopanib is a tyrosine kinase inhibitor that targets ABL1(T315I), as supported only by the preclinical evidence provided by DTC (Figure 7).

#### C-SPADE

**Figure 8:**
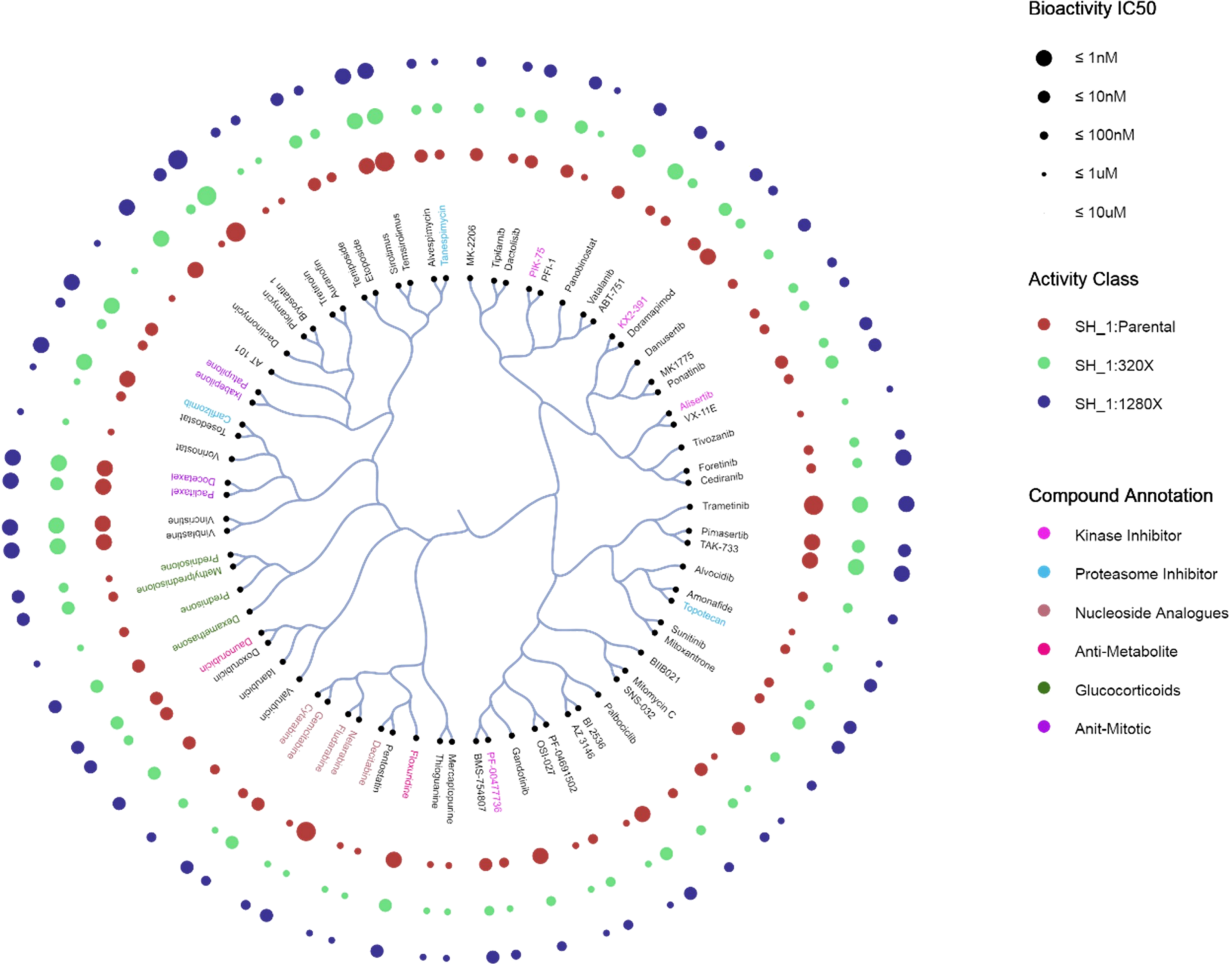
Compound similarity-based clustering in an example bioactivity dataset with C-SPADE. The dataset contains 75 compounds across three types of cell lines, with a subset of compounds annotated by the inhibitor type using different color codes as shown in the legend. Bubble size represents five potency classes in terms of IC50, which are color coded for different activity classes.

 C-SPADE (http://cspade.fimm.fi/) is an exploratory web application that provides interactive visualization of drug-profiling assays based on compound-centric similarity clustering (43). C-SPADE can visualize both cell/sample-specific compound sensitivity bioactivity data as well as protein/target-specific compound–target bioactivity data, such as those extracted from DTC. It allows the users to adjust multiple parameters in the clustering procedure, including fingerprints that are used to compute structural similarities between the compounds (default is ECFP4 fingerprint). Users can, for instance, export bioactivity data from DTC and obtain high-quality compound clustering-based visualizations through C-SPADE, as shown in Figure 8, with the aim to highlight new compound candidates for drug-repurposing applications. For instance, both TAK-733 (investigational compound) and trametinib (approved for thyroid cancer) are clustered together in Figure 8. Based on their bioactivity profiles from DTC, both are potent against mitogen-activated protein kinases. TAK-733 shows sensitivity in melanoma cancer cell lines, and trametinib has also been tested for melanoma in late trials, suggesting a potential efficacy of trametinib also in melanoma.

## Conclusions and future perspectives

In the recent years, multiple resources have been developed based on diverse compound collections to define primary targets for small molecules and identify potent molecular probes for specific molecular targets (17). While these resources have been useful for phenotypic profiling and drug-development efforts, they provide only a limited assay annotation for the end users to understand and sort out the variability in the bioactivity data that are typically generated using phenotypic assays. Moreover, the existing data curation is largely being done in a closed manner, lacking an open and transparent platform that would allow community-level participation. To address this issue, we recently launched DTC, a crowdsourcing web platform that aims to standardize the collection, management, curation and annotation of the notoriously heterogeneous compound–target bioactivity data to facilitate drug discovery, target identification and drug repurposing (25).

Since its original release, the number of assay annotations has vastly increased, and we have made significant improvements to extend the utility of the DTC database even further. Firstly, a comprehensive, new bioactivity dataset is now integrated from BindingDB. Similar to DTC, BindingDB is a frontend user portal to list drug–target interactions, but unlike DTC, it does not support crowdsourcing nor provides the assay information necessary for biologically meaningful and mechanistically relevant drug classification, ranking and clustering. Secondly, in the current release of DTC (version 2.0), we have integrated clinical trial information and disease–gene associations to support especially drug-repositioning applications. Users can, for the first time, extract target and μBAO assay data and combine it with diseases and/or mutant-specific information for oncology application. This new level of information is useful in identifying, clustering and ranking compounds based on translational potential, potentially facilitating clinical decision-making. Lastly, we also anticipate that the availability of full database dump and comprehensive API will increase the reusability of DTC data in many clinical and biological applications. These features highlight the open-access concept of DTC, which promotes drug discovery and extends the utility of drug annotations for new applications, as demonstrated with the two use cases and two built-on application tools.

We hope that the integration of new data resources and improvements in the DTC platform will further attract the community to join this crowdsourcing effort. With computational biology finally demonstrating a potential for guiding researcher-driven validation in the laboratory for translational applications (44), we fully expect that creative end users can find new and clinically meaningful ways of harnessing the compound–target data collected in DTC to come up with novel approaches how small molecules can be used in both research and clinics.

## Supplementary Material

Supplementary Data

## Notes

**Database URL**: https://drugtargetcommons.fimm.fi/
